# Psychosocial Distress Scores and Needs among Newly Diagnosed Sarcoma Patients: A Provincial Experience

**DOI:** 10.1155/2019/5302639

**Published:** 2019-07-01

**Authors:** Amirrtha Srikanthan, Bonnie Leung, Aria Shokoohi, Alannah Smrke, Alan Bates, Cheryl Ho

**Affiliations:** ^1^Division of Medical Oncology, The Ottawa Hospital Cancer Centre, Ottawa, Ontario K1H 8L6, Canada; ^2^Medical Oncology, BC Cancer, Vancouver Centre, Vancouver, British Columbia V5Z4E6, Canada; ^3^Psychosocial Oncology, BC Cancer, Vancouver Centre, Vancouver, British Columbia V5Z4E6, Canada

## Abstract

**Introduction:**

Information on the psychosocial distress and needs of sarcoma patients at diagnosis is sparse. The Canadian Problem Checklist (CPC) and Psychosocial Screen for Cancer-Revised (PSSCAN-R) are validated tools to identify cancer patients' distress and are administered to all new patients referred to BC Cancer prior to their consultation. We used the CPC and PSSCAN-R to understand sarcoma patients' needs at the initial oncology consultation in British Columbia, Canada.

**Materials and Methods:**

All sarcoma patients who completed the CPC and PSSCAN-R within 6 months of diagnosis between 2011 and 2016 were included. The retrospective chart review identified baseline demographics: age, performance status, disease location, resectability, and histology. Analysis was conducted using descriptive statistics, chi-squared test, Fisher's exact test, and Kaplan–Meier method.

**Results:**

413 sarcoma patients were identified. The majority of patients were over the age of 40 (83.3%) with ECOG performance status 0-1 (82.6%) and lower extremity tumors (55.4%). The most common diagnoses were liposarcoma 21.3%, undifferentiated pleomorphic sarcoma 12.1%, and myxofibrosarcoma 11.1%. At the initial consultation, 42.6% of patients were deemed resectable, 8.5% unresectable/metastatic, and 48.9% required further staging investigations. The top three patient-reported distress symptoms were feeling tense and unable to relax (50%), feeling nervous and shaky (48%), and experiencing repetitive and scary thoughts (42%). 38% of patients had subclinical/clinical anxiety symptoms, and 21% of patients had subclinical/clinical depression symptoms. 5% of patients expressed suicidal ideation. The top three concerns/needs were understanding of illness/treatment (45.5%), fear/worries (45.3%), and worry about family (23%). No differences in overall survival were identified for patients displaying symptoms of depression or anxiety versus no symptoms.

**Discussion:**

Up to 45% of sarcoma patients experience some form of psychological distress at disease presentation. Patients desire information about their diagnosis and treatment. Tailored interventions to individual psychological comorbidity and improved patient education resources would be beneficial.

## 1. Introduction

Depression and anxiety are common psychological comorbidities among cancer patients [1–3]. The impact of psychological distress has been increasingly associated with poor adherence to therapy, quality of life, and cancer prognosis [2, 4–6]. In addition, patients with cancer often have unmet psychosocial needs, and their desire for information is well documented with international recommendations advocating for needs and distress screening [7]. Programs that routinely screen for and treat distress have been shown to be feasible and have meaningful impact on distress levels [7, 8]. Evidence further suggests that unmet psychosocial needs may also correlate with higher depression and anxiety scores in the setting of cancer [9, 10].

Despite recognizing the morbidity of psychosocial distress, there is limited information regarding psychological comorbidity and psychosocial needs among sarcoma patients soon after diagnosis [11–14]. This evidence vacuum is concerning as early identification of distress can lead to timely treatment and improved outcomes [7, 8, 10]. Sarcomas are a rare group of mesenchymal origin tumors which annually represent 1% of all malignancies in adults [15]. This rarity results in limited high quality information available to patients [16] and limited treatment-specific information for different sarcoma histologies [17]. It is therefore anticipated that individuals diagnosed with sarcoma may potentially experience greater informational needs and psychosocial distress around diagnosis.

The Canadian Problem Checklist (CPC) was developed by the Screening for Distress Toolkit Working Group, through the Canadian Partnership Against Cancer, to screen for the most common, evidence-based concerns experienced by patients [8, 18]. It assesses cancer patient distress in six key domains: emotional, practical, spiritual, social, informational, and physical. The Psychosocial Screen for Cancer-Revised (PSSCAN-R) questionnaire identifies at-risk individuals who require timely psychosocial intervention. The CPC and PSSCAN-R are validated tools to identify cancer patients' distress and needs [19, 20]. Both questionnaires are routinely administered to all new patients referred to BC Cancer at the initial consultation, prior to being seen by a physician. The physician and treating medical team review the responses to tailor discussions and support to individual patient needs (Supplementary Figure 1).

Here, we describe the preconsultation psychosocial distress scores and needs of newly diagnosed sarcoma patients in a provincial cohort, using the CPC and PSSCAN-R, to characterize patient-reported needs and psychological distress at diagnosis.

## 2. Materials and Methods

All sarcoma patients who attended BC Cancer from April 2011 to 2016 and prospectively completed the validated CPC and PSSCAN-R questionnaire at the time of their first visit were evaluated. BC Cancer is a provincial institution with six specialty cancer centers distributed throughout the province, serving a population of 4.6 million ranging from remote to urban [21]. All sarcoma patients are assessed at BC Cancer and reviewed at a provincial multidisciplinary board of sarcoma specialists. BC Cancer delivers all radiotherapy within the province and the majority of systemic therapy. All patients who are seen at BC Cancer are asked to complete the Health Assessment Form which includes the PSSCAN-R and have an additional 15 minutes booked to their initial consultation to accommodate completion of the questionnaire. The number of patients who have not completed the questionnaire is deemed to be low due to this process; however, the actual denominator is unknown.

Patient records were extracted from provincial databases [22]. First, consultation visits were undertaken within 6 months of pathological diagnosis. The time frame of 6 months was chosen as some patients may undergo surgery prior to referral to BC Cancer. In order to capture as many sarcoma patients as possible, a 6-month window was selected. This 6-month window also remains within the adjustment period for a cancer diagnosis [23, 24].

Gastrointestinal stromal tumors (GIST) were excluded, as this subtype of sarcoma has more information and treatment options available than other histologies. Baseline demographics including age, performance status (PS), location of disease, resectability of disease and histology, were collected by the retrospective chart review by three independent abstracters (Bonnie Leung, Aria Shokoohi, and Alannah Smrke), and disagreement was resolved through consensus. PS was measured by the Eastern Cooperative Oncology Group (ECOG) scale and was rated by clinicians at the time of screening. A score of 0 to 1 was defined as good PS and 2 or greater as poor PS.

### 2.1. Distress Scores

Psychosocial depressive and anxiety symptoms were collected using the PSSCAN-R. The anxiety and depression subscales consist of questions on a Likert scale (0 (no symptoms) to 4 (greatest symptoms)), regarding the patient's level of anxiety and depression. The full questionnaire is available in Supplementary Figure 1. Responses on the PSSCAN-R were evaluated to determine the presence of symptoms of depression or anxiety as per previously validated methods [19, 20]. The questionnaire has been validated against the Hospital Anxiety and Depression Score (HADS) [19, 20]. Scores less than 8 indicate minimal symptoms, 8 to 10 indicate subclinical symptoms of depression or anxiety, and scores of 11 or greater are suggestive of severe symptoms.

### 2.2. Canadian Problem Checklist

The CPC was developed by the Screening for Distress Toolkit Working Group, through the Canadian Partnership Against Cancer, to screen for the most common, evidence‐based concerns experienced by patients [8, 18]. The CPC identifies distress in six domains: emotional, practical, spiritual, social, informational, and physical (Supplementary Figure 1). The tool requests the following: “Please check all of the following items that have been of concern or a problem for you in the past week including today.” Multiple response options exist within each domain. For example, in the practical domain, the following options are available: (1) work/school; (2) finances; (3) getting to and from appointments; (4) accommodation.

### 2.3. Perceived Social Support

The social support assessment consists of five dichotomous items derived from a modified Social Network and Support Assessment tool used in the Epidemiological Study of the Elderly [25]. The patients are asked whether they live alone, have lost a life partner, can receive assistance with their instrumental activities of daily living (IADLs) when needed, have emotional support, and have regular contact with friends and family.

### 2.4. Statistical Analysis

Descriptive statistical analyses were utilized, and frequency of occurrence and percentage was calculated for each of the independent variables. Univariate analysis using the chi-squared test and Fisher's exact test was conducted to compare groups based on gender, age, performance status, resectability of disease, and location of primary. Overall survival (OS) was estimated using the Kaplan–Meier method with log rank comparison. OS was calculated from diagnosis until death. Patients were censored for the OS outcome on the date of the last follow-up or investigation, indicating that they were alive. All analyses were performed using IBM SPSS Statistics software, version 25 (SPSS Institute Inc., Cary, NC). Statistical significance was defined using a two-tailed *p* value of <0.05.

### 2.5. Ethical Approval

The British Columbia Cancer Agency Ethics Board (now renamed the BC Cancer Ethics Board) and privacy office approved this study prior to commencement (University of British Columbia-BC Cancer Research Ethics Board; H16-01047). Consent was not obtained from individual patients due to the retrospective study design; however, all patient information was anonymized and deidentified by the investigators for analysis.

## 3. Results

### 3.1. Baseline Characteristics

413 sarcoma patients were identified in our cohort, and baseline characteristics are available in Table 1. In our study, 62% of patients completed the questionnaire within 1 month of diagnosis and 87% within 2 months, and the median time to completion was 0.72 months.

The median age was 61 years of age (range: 18–95 years). 52.1% were male, and the majority of patients had good PS ECOG 0-1 (82.6%). Location of sarcoma was predominantly lower extremity (55.4%). The most common diagnoses were liposarcoma (21.3%), undifferentiated pleomorphic sarcoma (12.1%), and myxofibrosarcoma (11.1%). At the time of the initial consultation, 42.6% of patients were deemed resectable; however, 48.9% of patients required further staging investigations. The majority of patients did not live alone (80%) and had access to assistance for instrumental activities of daily living at the time of consultation (87%). 20% of patients had depressive symptoms 2 years prior to diagnosis.

### 3.2. Psychosocial Distress Scores

Two weeks prior to consultation, up to 50% of patients experienced the presence of a distress symptom on the PSSCAN-R, as presented in Table 2. The top four patient-reported symptoms on PSSCAN-R were the presence of feeling tense and unable to relax (50%), feeling nervous and shaky (48%), experiencing repetitive and scary thoughts (42%), and feeling restless and unable to sit still (37%). Excluding the lowest severity (“a little bit”) on the Likert scale, the most frequent patient-reported symptoms were feeling nervous and shaky (16.7%), feeling tense and unable to relax (15.5%), and feeling restless and unable to sit still (14.3%).

Of the entire cohort, 17/413 individuals did not answer psychosocial distress questions. 44.7% of the entire cohort had distress at the initial consultation: 23.5% of patients had symptoms of subclinical or clinical anxiety alone, 5.5% had symptoms of subclinical or clinical depression alone, and 15.7% had symptoms of both anxiety and depression. Suicidal ideation was present in 5% of patients.

Female patients reported more symptoms of anxiety (50.5% vs 28.6%, *p* < 0.001) and symptoms of depression (25.8% vs 17%, *p*=0.032) compared to males. Patients with poorer PS (ECOG 2 or greater) reported more anxiety (32% vs 16%, *p*=0.010) and depression (18% vs 7%, *p*=0.002) than fitter patients. No difference in anxiety or depression distress scores was identified for patients below 40 years of age versus older patients, resectable versus unresectable disease, or location of primary.

### 3.3. Psychosocial Needs of Newly Diagnosed Sarcoma Patients

**Figure 1:**
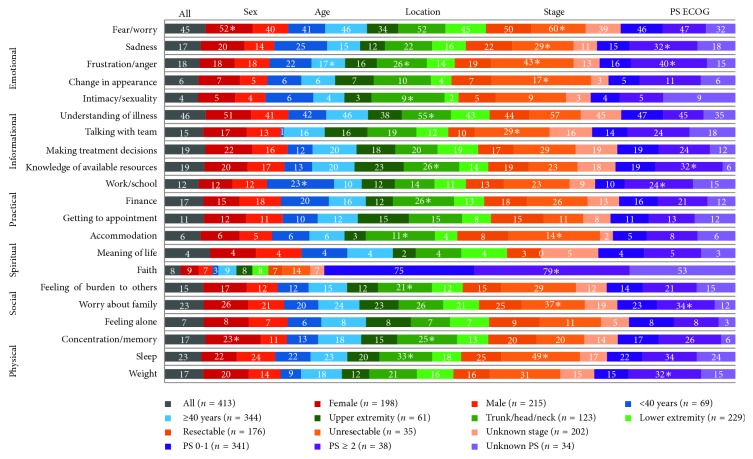
Reported concerns on the Canadian Problem Checklist by patient subgroups. The numbers represent the percentage of patients that reported the concern as positive. PS = performance status; ECOG = Eastern Cooperative Oncology Group; ^*∗*^statistically significant with a *p* value <0.05.

The top five concerns among all newly diagnosed patients were understanding of one's illness/treatment (45.5%), fear/worries (45.3%), worry about family (23%), sleep (23%), and making treatment decisions (18.9%) (Figure 1, Supplementary Table 1). Lowest ranking concerns were meaning of life (3.9%), sexuality (4.1%), appearance (5.8%), feeling alone (7.3%), and faith (7.7%).

Compared to men, women endorsed greater emotional concerns (74.7% vs. 68%, *p* = 0.003) and specifically reported greater fear/worry (51.5% vs. 45.3%, *p* = 0.009). Women also reported greater difficulty concentrating (23.2% vs. 16.9%, *p* = 0.002).

Among patients with poor PS (ECOG 2 or greater), there were higher rates of sadness (31.6% vs. 15.2%, *p* = 0.039) and frustration/anger (39.5% vs. 16.1%, *p* = 0.002) and greater concerns about available resources (31.6% vs. 18.5%, *p* = 0.020), work/school (23.7% vs. 10.3%, *p* = 0.046), and weight (31.6% vs. 15.2%, *p* = 0.036), compared to fitter patients.

Compared to older patients, adolescents and young adults (aged 39 years and less, AYA) reported more concerns about work/school (23.2% AYA vs. 9.6% non-AYA, *p*=0.003). No other domains were significantly different; however, trends were seen in weight (8.7% AYA vs. 18.3% non-AYA, *p*=0.053), sadness (24.6% AYA vs. 15.4% non-AYA, *p*=0.078), and making treatment decisions (11.6% AYA vs. 20.3% non-AYA, *p*=0.095).

Patients with unresectable disease more commonly reported fear/anxiety (60.0% vs. 45.3%, *p*=0.016), emotional sadness (28.6% vs. 16.9%, *p*=0.003), frustration/anger (42.9% vs. 18.2%, *p*=0.001), emotional changes with appearance (17.1% vs. 5.8%, *p*=0.009), challenges communicating with the health care team (28.6% vs. 14.8%, *p*=0.019), practical concerns regarding accommodation (14.3% vs. 5.6%, *p*=0.003), worry about family (37.1% vs. 23.0%, *p*=0.049), and greater difficultly sleeping (48.6% vs. 23.0%, *p* < 0.001).

Individuals with trunk/head and neck tumors reported greater frustration and anger (26% vs. 18.2%, *p*=0.025), more intimacy/sexuality concerns (8.9% vs. 4.1%, *p*=0.005), greater difficulty understanding their illness (54.5% vs. 45.5%, *p*=0.046), lesser knowledge of available resources (26% vs. 18.6%, *p*=0.011), and greater difficulties with finances (26% vs. 16.5%, *p*=0.003). These individuals were also more likely to feel like a burden to others (21.1% vs. 14.5%, *p*=0.046) and reported more physical symptoms (concentration/memory impairment: 25.2% vs. 16.9%, *p*=0.014; poor sleep: 33.3% vs. 23.0%, *p*=0.005).

### 3.4. Survival

The 5-year overall survival (OS) for the whole cohort was 65.4%. The 5-year OS by minimal symptoms versus subclinical/clinical symptoms of anxiety was 63.8% versus 67.4% (*p*=0.551). The 5-year OS by minimal symptoms versus subclinical/clinical symptoms of depression or anxiety was 66.8% versus no symptoms 60.8% (*p*=0.526).

## 4. Discussion

In this provincial cohort of consecutive sarcoma patients over 5 years, up to 45% of all sarcoma patients experience some form of significant psychological distress at the initial oncology consultation, with higher distress scores identified in female patients and those with poor performance status. Consistent with the relative lack of information available to sarcoma patients and the uncertainty that it generates, understanding of illness/treatment and fear/worries rank as the top two concerns of all patients.

This study represents the largest cohort of sarcoma patients for whom psychosocial data are available. These findings differ with the previously published findings of 142 sarcoma patients, of which 49 were in the diagnostic phase, assessed in Portugal [11, 14]. Although the Portuguese study used the Portuguese version of the HADS, we have used a measure validated against the HADS [20], enabling comparability. Similar to the Portuguese study, we identified clinically significant distress scores and different distress scores based on gender [11]. However, our larger cohort identified no difference between younger and older patients as the Portuguese study did.

Additional work by the Portuguese group assessed 36 sarcoma patients at the time of diagnosis and again during treatment for emotional distress using the HADS. In this work, 52.8% and 66.7% of patients experienced nonclinical anxious and depressive symptoms, respectively, and 25% and 11.1%, respectively, continued to experience clinical anxiety and depression after treatment [13]. These proportions vary compared to our cohort in which 38% and 21% experienced subclinical and clinical anxiety and depression, respectively. A study of 76 extremity sarcoma patients at diagnosis from Australia identified that 22% and 15% of the patients experienced symptoms of anxiety and depression, respectively [26]. No significant differences were identified in our cohort for the location of tumor. Variations between these studies may be due to differences in methods, such as scales used, cultural differences, and differences in cohort demographics.

Limited studies have been undertaken in a North American context; however, a small study of 11 patients from Pennsylvania, Philadelphia, assessed patients at diagnosis and then during the first twenty-one days of chemotherapy treatment using the Edmonton Symptom Assessment Scale and the Functional Assessment of Cancer Therapy-General [12]. Although this study assessed quality of life as opposed to psychosocial needs and distress, anxiety and depression were among the most commonly reported symptoms [12].

Of concern, in our cohort, 5% of patients endorsed suicidal ideation. The practice at our institution includes same day social worker/counsellor/psychiatrist assessments for those with suicidal ideation to mitigate suicide attempts. US population data indicate that the overall suicide incidence in sarcoma patients is three times greater than the age-, race-, and gender-adjusted US general population suicide incidence of 13 per 100,000 person-years [27]. A higher sarcoma suicide incidence was observed in men, patients of white race, patients 21 to 30 years old and 61 to 70 years old, patients with cancer of the vertebral column and pelvic bones, and patients within the first 5 years of cancer diagnosis [27]. These characteristics can be considered for identifying at-risk patients during psychosocial distress screening with the hope of reducing suicide incidence.

With respect to psychosocial needs at diagnosis, concerns around meaning of life, faith, intimacy/sexuality, and appearance ranked lowest in our cohort. This finding highlights the informational needs at the initial consultation are different than that indicated by survivorship data where sexuality and appearance emerge as common survivorship issues and concerns among sarcoma patients [28–30]. The initial concerns of sarcoma patients relate to survival and getting though treatment at the initial consultation, which is corroborated by the top concerns such as understanding of illness/treatment, fear/worries, worry about family, and making treatment decisions. The development of high-quality patient education materials can assist with this need, in particular as resources are less available for rare tumors.

Variation in psychosocial needs was seen in different subgroups of patient. Younger patients (39 years of age and under), for example, reported higher concerns around work and school compared to the entire cohort. Head and neck patients reported greater concerns in finances, sexuality/intimacy, and accessing information. Patients with unresectable disease demonstrated higher proportions of emotional needs around addressing depressive symptoms, anger, frustration, and anxiety. Such variations highlight the importance of tailoring resources to address individual patient needs. Targeted support interventions may also contribute to alleviating psychological comorbidity [9, 10], and thus, the development of such resources can potentially minimize persistent depression or anxiety.

Compared to other tumor groups treated in the province, comparable rates of distress were identified with screening by the same tool. In lung cancer, for example, up to 45% of patients reported subclinical or clinical symptoms of anxiety, whereas up to 31% of patients reported subclinical or clinical depression symptoms [21]. In a 26,000 cohort of all cancer patients in the province aged 65 years and over, rates of subclinical or clinical symptoms of anxiety ranged from 21–45% and rates of subclinical or clinical depression symptoms ranged from 25–46% depending on the age bracket [31]. Thus, this tool is generalizable to a various tumor types and ages.

Natural next steps of this work are to assess changes in information need and distress scores over time. Both questionnaires are currently used in clinical practice to guide clinicians regarding a patient's initial concerns. Routine implementation of this tool during the continuum of care will ensure patients' changing needs are identified. The results of this tool are currently used to guide resource development within the psychosocial oncology department. Expanding usage during treatment will ensure ongoing early identification of distress. Additionally, based on the responses from the tool, clinical practice has been impacted by the development of additional institutional resources to assist patients, such as the Young Adult Art Therapy support group and the job search program [32].

In this cohort, up to 45% of patients have significant distress symptoms at the initial consultation, consistent with prior studies that demonstrate up to 40% of patients with cancer may suffer from psychological distress [33]. Thus, the rates identified in this sarcoma cohort appear comparable to other tumor sites. However, whether this distress is part of the spectrum of the natural/normal reaction to a diagnosis of cancer or potential presentation of a new comorbidity is unknown. A Dutch cohort study of 533 sarcoma patients over 8 years identified that the incidence of depression after diagnosis differed significantly from cancer-free controls, and sarcoma patients were more likely to develop depression [34]. Whether early identification of depressive symptoms and psychosocial needs with concomitant early interventions affects this comorbidity is unknown.

Although this work presents new psychosocial data on the largest cohort of sarcoma patients to date, this study has limitations. The questionnaire is offered to all new patients; however, the level of reporting is patient determined; thus, it may not fully capture the degree of distress. Given it is administered only at diagnosis, we are unable to assess the evolution of psychosocial distress and needs over the treatment trajectory. Physical symptoms are not captured on this screening questionnaire which might further impact distress scores. Due to small numbers, variations in distress between sarcoma subtypes could not be assessed. Additionally, the tool does not capture causes of fear/worries; however, flags the concern to ensure clinicians inquire about this during the visit. Current oncology treatments and physical symptom data are not available, which if available, could improve the delivery of psychosocial care throughout the treatment trajectory. To improve uptake and reporting, it may be helpful to emphasize to patients the use of the questionnaires in developing patient-centred programming. This longitudinal aspect is being piloted for brain cancer patients at the institution and may be considered for sarcoma. During this pilot, repeat administration of the anxiety and depression component of the PSSCAN-R questionnaire is undertaken. Patients are provided with an electronic device while waiting for their physician's appointments to complete the questionnaire. The information is sent electronically to the Patient and Family Counselling department in real time so that services can be appropriately mobilized if patients note significant distress. The effectiveness of this tool and the evolution of distress will be examined once the pilot is complete.

## 5. Conclusions

Despite these limitations, our study demonstrates the prevalence of anxiety and depression at the initial consultation in a large cohort of newly diagnosed sarcoma patients and characterizes the most common psychosocial needs of sarcoma patients. This information is valuable for the program development of sarcoma patients internationally. The psychosocial needs identified highlight the importance of providing understandable information upfront and assist with treatment decision-making. Patient characteristics such as site of primary disease, age, and gender are important considerations for supportive psychosocial program development. Ongoing work to assess needs through the treatment trajectory can assist in further refining program development and resource administration.

## Figures and Tables

**Table 1:** Baseline characteristics.

		Total (*n* = 413)
Age	Median (years)	61
	Interquartile range (years)	46.5–73
	<40 years	69 (16.7%)
	>40 years	344 (83.3%)

Sex	Female	198 (47.9%)
	Male	215 (52.1%)

Stage at screening	Resectable	176 (42.6%)
	Unresectable/metastatic	35 (8.5%)
	Unknown	202 (48.9%)

Location	Upper extremity	61 (14.8%)
	Trunk or head and neck	123 (29.8%)
	Lower extremity	229 (55.4%)

Histology	Alveolar soft part sarcoma	1 (0.2%)
	Angiosarcoma	8 (1.9%)
	Ewing sarcoma	5 (1.2%)
	Fibrosarcoma	3 (0.7%)
	Giant cell tumor	46 (11.1%)
	Leiomyosarcoma	45 (10.9%)
	Liposarcoma	88 (21.3%)
	Malignant peripheral nerve sheath tumor	11 (2.7%)
	Myxofibrosarcoma	46 (11.1%)
	Osteosarcoma	4 (1.0%)
	Perivascular epithelioid cell tumor	1 (0.3%)
	Rhabdomyosarcoma	6 (1.5%)
	Synovial sarcoma	15 (3.6%)
	Undifferentiated pleomorphic sarcoma	50 (12.1%)
	Soft tissue sarcoma, not otherwise specified	84 (20.3%)

ECOG at screening^*∗*^	0-1	341 (82.6%)
	≥2	38 (9.2%)
	Unknown	34 (8.2%)

Lives alone^*∗*^	Yes	81 (20%)
	No	323 (78%)
	Missing	9 (2%)

Requires assistance^*∗*^	Yes	361 (87%)
	No	38 (9%)
	Missing	14 (3%)

Regular contact with loved ones^*∗*^	Yes	395 (96%)
	No	10 (2%)
	Missing	8 (2%)

Widowed^*∗*^	Yes	34 (8%)
	No	364 (88%)
	Missing	15 (4%)

Has emotional support^*∗*^	Yes	387 (94%)
	No	14 (3%)
	Missing	12 (3%)

^*∗*^Self-reported by respondents in questionnaire.

**Table 2:** Psychosocial distress findings among newly diagnosed sarcoma patients (*n* = 413).

	Not at all	A little bit	Moderately	Quite a bit	Very much	No answer
Past week heart races	299 (72%)	65 (16%)	16 (4%)	8 (2%)	4 (1%)	21 (5%)
Feels loss of self-control	311 (75%)	46 (11%)	21 (5%)	11 (3%)	4 (1%)	20 (5%)
Lost interest	297 (72%)	64 (16%)	13 (3%)	15 (4%)	1 (0.2%)	23 (6%)
Nervous	213 (52%)	107 (26%)	39 (9%)	24 (6%)	6 (1.5%)	24 (6%)
Tense	207 (50%)	119 (29%)	35 (9%)	25 (6%)	4 (1%)	23 (6%)
Scary thoughts	241 (58%)	92 (22%)	30 (7%)	19 (5%)	9 (2%)	22 (5%)
Restless	262 (63%)	70 (17%)	33 (8%)	19 (5%)	7 (2%)	22 (5%)
Suicidal thoughts	386 (94%)	5 (1%)	0 (0%)	0 (0%)	1 (0.2%)	21 (5%)
Depressed in the past 1 year	290 (70%)	61 (15%)	20 (5%)	12 (3%)	9 (2%)	21 (5%)
Depression in the past 2 years	330 (80%)	30 (7%)	12 (3%)	10 (2.5%)	8 (2%)	23 (6%)

## Data Availability

Data will be made available to interested parties through communication with the corresponding author. Data are currently held in a database which can be electronically accessed. Access to the data will comply with institutional research ethics board processes.

## Supplementary Materials

Supplementary Materials Supplementary Figure 1: BC Cancer Health Assessment Form which includes the Psychosocial Screen for Cancer-Revised (PSSCAN-R) and Canadian Problem Checklist (CPC). Supplementary Table 1: reported concerns on the Canadian Problem Checklist by patient subgroups, in numerical values.
