# Supplementary material for: Psychosocial Distress Scores and Needs among Newly Diagnosed Sarcoma Patients: A Provincial Experience
Source: Sarcoma. 2019 Jul 1;2019:5302639. doi: 10.1155/2019/5302639 (PMC6636442; doi:10.1155/2019/5302639)
Supplement: Supplementary Materials — Supplementary Figure 1: BC Cancer Health Assessment Form which includes the Psychosocial Screen for Cancer-Revised (PSSCAN-R) and Canadian Problem Checklist (CPC). Supplementary Table 1: reported concerns on the Canadian Problem Checklist by patient subgroups, in numerical values. [file 5302639.f1.pdf]

BC CANCER
Provincial Health Services Authority

PSSCAN-R Psychological Screening

Please answer the following questions to help us learn more about your well being. A serious illness can affect the quality of your life in many ways. We may contact you to offer our counselling services based on the information you provide to us, or contact you regarding opportunities to participate in research.

Part A:
Please respond to each question with "Yes" or "No" by making a circle around the appropriate answer. There are no right or wrong answers.

1.	Do you live alone?	No	Yes
2.	When you need help, can you count on anyone to help with daily tasks such as grocery shopping, cooking, giving you a ride?	No	Yes
3.	Do you have regular contact with friends or relatives?	No	Yes
4.	Have you lost your life partner within the last few years?	No	Yes
5.	Can you count on anyone to provide you with emotional support?	No	Yes

Part B:
Please check all of the following items that have been of concern or a problem for you in the past week including today.*

6. **Emotional:**<br>○ Fears/Worries<br>○ Sadness<br>○ Frustration/Anger<br>○ Changes in appearance<br>○ Intimacy/Sexuality	7. **Informational:**<br>○ Understanding my illness/treatment<br>○ Talking with the health care team<br>○ Making treatment decisions<br>○ Knowing about available resources
8. **Practical:**<br>○ Work/School<br>○ Finances<br>○ Getting to & from appointments<br>○ Accommodation	9. **Spiritual:**<br>○ Meaning/Purpose of life<br>○ Faith
10. **Social/Family:**<br>○ Feeling a burden to others<br>○ Worry about family/friends<br>○ Feeling alone	11. **Physical:**<br>○ Concentration/Memory<br>○ Sleep<br>○ Weight

Other concerns, please specify: ______________________________

* **Canadian Problem Checklist** developed by the Canadian Partnership Against Cancer, August 2010.

BC CANCER
Provincial Health Services Authority

Part C: Please place an 'X' in the box that best describes what you have experienced.

	Not at all	A little bit	Moderately	Quite a bit	Very much
12. **During the past week** I have felt my heart race and I tremble.					
13. **During the past week** I have felt that I cannot control anything.					
14. **During the past week** I have lost interest in things I usually cared for or enjoyed.					
15. **During the past week** I have felt nervous and shaky inside.					
16. **During the past week** I have felt tense and cannot relax.					
17. **During the past week** my thoughts are repetitive and full of scary things.					
18. **During the past week** I have felt restless and find it difficult to sit still.					
19. **I have *recently*** thought about taking my life. **NOTE: If you have, please speak with a member of your health care team and/or your family doctor today.**					
20. **In the past year**, I have had 2 weeks or during which I felt sad, blue or depressed.					
21. **I have had 2 years or more in my life** when I felt depressed or sad most days even if I felt okay sometimes.					

Thank you for taking the time to respond to this form.

If you or your family is currently struggling with the stress of your diagnosis, information and support is available on our website: www.bccancer.bc.ca/health-info/coping-with-cancer or by calling:
BC Cancer Patient & Family Counselling Departments

Abbotsford	604.851.4733
Kelowna	250.712.3963
Prince George	250.645.7330
Surrey	604.930.4000
Vancouver	604.877.6000 x 672194
Victoria	250.519.5525

Patient and Family Counselling Documentation:

D = ______________________ A = ______________________
Comments: ______________________
Reviewed by: ______________________
Date: ______________________

Revised April 2014 Review May 8, 2018

Supplementary Table 1: Reported Concerns on the Canadian Problem Checklist by Patient Subgroups

Patient reported experience at diagnosis	All	Gender: Female (N=198)	Gender: Male (N=215)	P value	Age: 39 years or less (N=69)	Age: 40 years or older (N=344)	P value	Location: Upper Extremity (N=61)	Location: Trunk/ Head and Neck (N=123)	Location: Lower extremity (N=229)	P value	Stage: Resectable (N=176)	Stage: Unresectable or metastatic (N=35)	Stage: Unknown (N=202)	P value	PS: ECOG 0-1 (N=341)	PS: ECOG 2 or greater (N=38)	PS: Unknown (N=34)	P value
EMOTIONAL																			
Fear/Worry	187 (45.3%)	102 (51.5%)	85 (39.5%)	0.018	28 (40.6%)	159 (46.2%)	0.428	21 (34.4%)	64 (52%)	102 (44.5%)	0.074	88 (50.0%)	21 (60.0%)	78 (38.6%)	0.016	158 (46.3%)	18 (47.4%)	11 (32.4%)	0.285
Sadness	70 (16.9%)	40 (20.2%)	30 (14.0%)	0.115	17 (24.6%)	53 (15.4%)	0.078	7 (11.5%)	27 (22%)	36 (15.7%)	0.155	38 (21.6%)	10 (28.6%)	22 (10.9%)	0.003	52 (15.2%)	12 (31.6%)	6 (17.6%)	0.039
Frustration/Anger	75 (18.2%)	36 (18.2%)	39 (18.1%)	0.991	15 (21.7%)	60 (17.4%)	0.395	10 (16.4%)	32 (26%)	32 (14.4%)	0.025	33 (18.8%)	15 (42.9%)	27 (13.4%)	0.001	55 (16.1%)	15 (39.5)	5 (14.7%)	0.002
Change in Appearance	24 (5.8%)	14 (7.1%)	10 (4.7%)	0.303	4 (5.8%)	20 (5.8%)	1.00	4 (6.6%)	12 (9.8%)	8 (3.5%)	0.055	12 (6.8%)	6 (17.1%)	6 (3.0%)	0.009	18 (5.3%)	4 (10.5%)	2 (5.9%)	0.423
Intimacy Sexuality	17 (4.1%)	9 (4.5%)	8 (3.7%)	0.805	4 (5.8%)	13 (3.8%)	0.502	2 (3.3%)	11 (8.9%)	4 (1.7%)	0.005	8 (4.5%)	3 (8.6%)	6 (3.0%)	0.337	12 (3.5%)	2 (5.3%)	3 (8.8%)	0.310
INFORMATIONAL																			
Understanding of Illness	188 (45.5%)	99 (50.5%)	89 (41.4%)	0.093	29 (42%)	159 (46.2%)	0.597	23 (37.7%)	67 (54.5%)	98 (42.8%)	0.046	77 (43.8%)	20 (57.1%)	91 (45.0%)	0.343	159 (46.6%)	17 (44.7%)	12 (35.3%)	0.447
Talking with Team	61 (14.8%)	33 (16.7%)	28 (13.0%)	0.332	7 (10.1%)	54 (15.7%)	0.27	10 (16.4%)	23 (18.7%)	28 (12.2%)	0.245	18 (10.2%)	10 (28.6%)	33 (16.3%)	0.019	46 (13.5%)	9 (23.7%)	6 (17.6%)	0.216
Making Treatment Decision	78 (18.9%)	43 (21.7%)	35 (16.3%)	0.168	8 (11.6%)	70 (20.3%)	0.095	11 (18%)	24 (19.5%)	43 (18.8%)	0.969	29 (16.5%)	10 (28.6%)	39 (19.3%)	0.268	65 (19.1%)	9 (23.7%)	4 (11.8)	0.427
Knowledge of Available Resources	77 (18.6%)	40 (20.2%)	37 (17.2%)	0.451	9 (13%)	68 (19.8%)	0.236	14 (23%)	32 (26%)	31 (13.5%)	0.011	33 (18.8%)	8 (22.9%)	36 (17.8%)	0.786	63 (18.5%)	12 (31.6%)	2 (5.9%)	0.020
PRACTICAL																			
Work/School	49 (11.9%)	23 (11.6%)	26 (12.1%)	0.881	16 (23.3%)	33 (9.6%)	0.003	7 (11.5%)	17 (13.8%)	25 (10.9%)	0.720	23 (13.1%)	8 (22.9%)	18 (8.9%)	0.069	35 (10.3%)	9 (23.7%)	5 (14.7%)	0.046
Finance	68 (16.5%)	30 (15.2%)	38 (17.7%)	0.509	14 (20.3%)	54 (15.7%)	0.374	7 (11.5%)	32 (26%)	29 (12.7%)	0.003	32 (18.2%)	9 (25.7%)	27 (13.4%)	0.151	56 (16.4%)	8 (21.1%)	4 (11.8%)	0.569
Getting to Appointment	47 (11.4%)	24 (12.1%)	23 (10.7%)	0.757	7 (10.1%)	40 (11.6%)	0.837	9 (14.8%)	19 (15.4%)	19 (8.3%)	0.088	27 (15.3%)	4 (11.4%)	16 (7.9%)	0.076	38 (11.1%)	5 (13.2%)	4 (11.8%)	0.931
Accommodation	23 (5.6%)	12 (6.1%)	11 (5.1%)	0.831	4 (5.8%)	19 (5.5%)	1.00	2 (3.3%)	13 (10.6%)	8 (3.5%)	0.016	14 (8.0%)	5 (14.3%)	4 (2.0%)	0.003	18 (5.3%)	3 (7.9%)	2 (5.9%)	0.798
SPIRITUAL																			
Meaning of Life	16 (3.9%)	7 (3.5%)	9 (4.2%)	0.802	3 (4.3%)	13 (3.8%)	0.738	1 (1.6%)	5 (4.1%)	10 (4.4%)	0.613	6 (3.4%)	0 (0.0%)	10 (5.0%)	0.178	13 (3.8%)	2 (5.3%)	1 (2.9%)	0.869
Faith	32 (7.7%)	18 (9.1%)	14 (6.5%)	0.361	2 (2.9%)	30 (8.7%)	0.137	5 (8.2%)	8 (6.5%)	19 (8.3%)	0.827	12 (6.8%)	5 (14.3%)	15 (7.4%)	0.376	256 (75.1%)	30 (78.9%)	18 (52.9%)	0.015
SOCIAL																			
Feeling of Burden to Others	60 (14.5%)	34 (17.2%)	26 (12.1%)	0.163	8 (11.6%)	52 (15.1%)	0.575	7 (11.5%)	26 (21.1%)	27 (11.8%)	0.046	26 (14.8%)	10 (28.6%)	24 (11.9%)	0.056	47 (13.8%)	8 (21.1%)	5 (14.7%)	0.483
Worry about Family	95 (23.0%)	51 (25.8%)	44 (20.5%)	0.242	14 (20.3%)	81 (23.5%)	0.640	14 (23%)	32 (26%)	49 (21.4%)	0.618	44 (25.0%)	13 (37.1%)	38 (18.8%)	0.049	78 (22.9%)	13 (34.2%)	4 (11.8%)	0.077
Feeling Alone	30 (7.3%)	16 (8.1%)	14 (6.5%)	0.574	4 (5.8%)	26 (7.6%)	0.801	5 (8.2%)	8 (6.5%)	17 (7.4%)	0.908	16 (9.1%)	4 (11.4%)	10 (5.0%)	0.184	26 (7.6%)	3 (7.9%)	1 (2.9%)	0.597
PHYSICAL																			
Concentration/Memory	70 (16.9%)	46 (23.2%)	24 (11.2%)	0.002	9 (13%)	61 (17.7%)	0.385	9 (14.8%)	31 (25.2%)	30 (13.1%)	0.014	35 (19.9%)	7 (20.0%)	28 (13.9%)	0.260	58 (17.0%)	10 (26.3%)	2 (5.9%)	0.070
Sleep	95 (23.0%)	43 (21.7%)	52 (24.2%)	0.561	15 (21.7%)	80 (23.3%)	0.876	12 (19.7%)	41 (33.3%)	42 (18.3%)	0.005	44 (25.0%)	17 (48.6%)	34 (16.8%)	<0.001	74 (21.7%)	13 (34.2%)	8 (23.5%)	0.220
Weight	69 (16.7%)	39 (19,7%)	30 (14.0%)	0.146	6 (8.7%)	63 (18.3%)	0.053	7 (11.5%)	26 (21.1%)	36 (15.7%)	0.213	28 (15.9%)	11 (31.4%)	30 (14.9%)	0.076	52 (15.2%)	12 (31.6%)	5 (14.7%)	0.036

ECOG, Eastern Cooperative Oncology Group; PS, performance status
